# Closed-loop circuit for reduce oxygen waste on hollow-fiber oxygenators during extracorporeal technologies

**DOI:** 10.1186/s13054-021-03514-8

**Published:** 2021-02-25

**Authors:** Ignazio Condello, Flavio Rimmaudo, Giuseppe Speziale

**Affiliations:** 1 Department of Cardiac Surgery, Perfusion Service, Anthea Hospital, GVM Care and Research, Via Camillo Rosalba 35/37, 70124 Bari, Italy; 2 Department of Interventional Cardiology, Anthea Hospital, GVM Care and Research, Bari, Italy

Closed-loop systems are designed to dynamically regulate a given variable around a desired set point. Examples thereof surround our everyday lives, from cruise control maintaining the correct speed on the highway, to auto-pilot flying modern airplanes safely [1]. Currently, there are two groups of hollow-fiber membrane oxygenator used in practice. The first types are diffusion, plasma-resistant oxygenators that have been increasingly used for extracorporeal life support or extracorporeal membrane oxygenation for patients who can no longer be supported by mechanical ventilation. The second types are hollow-fiber membranes made of microporous poly-propylene that have been widely used for standard cardiopulmonary bypass (CPB) [2]. Microporous hollow-fiber membranes are primarily used for short-term cardiopulmonary bypass application, whereas non-microporous hollow-fiber membranes are primarily used for extracorporeal membrane oxygenation application (ECMO) [3]. During the use of the oxygenator, the lung membrane uses about 30% of the medical oxygen delivered by the gas mixer, and the remainder comes out of the gas exhaust and is dispersed into the environment. In this context, we present a closed system aimed at recovering oxygen from the gas outlet of the oxygenator. First, the system recovers through a disposable polymer tube the gas flow from the oxygenator outlet; second, the condensate and water vapor will be removed with a water separator; third, the CO_2_ will be removed through a soda lime container; fourth, an electric control unit will decide whether to enrich the% of oxygen recovered through an oxygen source, in relation to the percentage of FiO_2_ set on the gas mixer (Fig. 1). The closed-loop circuit for oxygenators is a “concept development,” and no data are provided on feasibility in this context. The system should be implemented with continuous monitoring to avoid:

- the water separator saturation, through excess water evacuation and appropriate filters
- the risk of increasing pressure in the gas outlet through a safety valve
- the risk for “rebreathing” and hence CO_2_ accumulation.

**Fig. 1:**
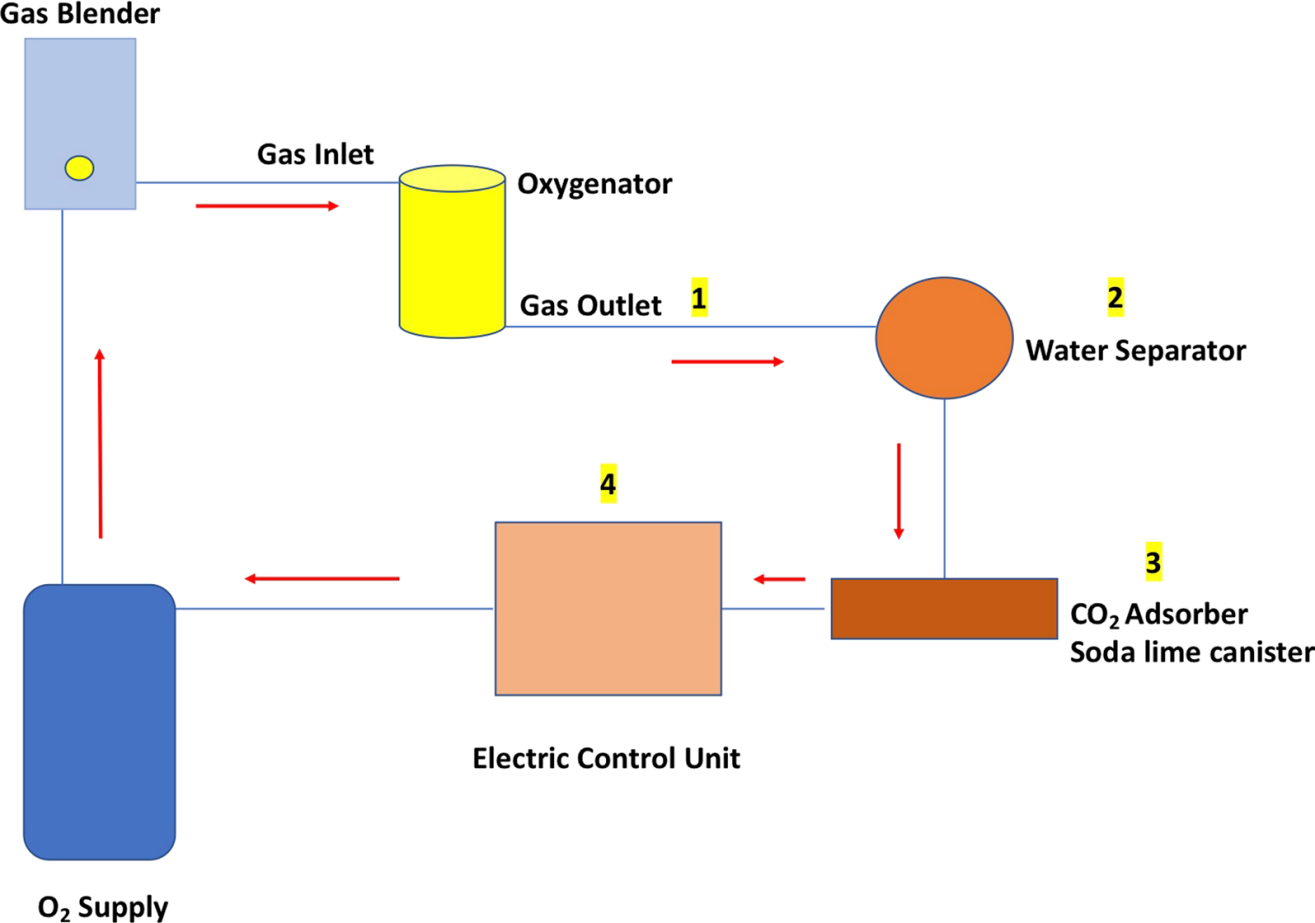
Closed-loop circuit for reduce oxygen waste on hollow-fiber oxygenators during extracorporeal technologies

Our proposal has the objective of reducing waste and optimizing the use of medical oxygen; at the same time, this closed system is crucial during the transport phases (mostly airplane), in particular for patients on ECMO, to maximize the use of oxygen, guaranteeing greater autonomy.

## Data Availability

Not applicable.
